# 1-(2,6-Dichloro­phen­yl)indolin-2-one

**DOI:** 10.1107/S1600536809005807

**Published:** 2009-02-25

**Authors:** Muhammad Hanif, Muhammad Rafiq, Muhammad Saleem, Ghulam Qadeer, Wai-Yeung Wong

**Affiliations:** a Department of Chemistry, Quaid-i-Azam Univeristy, Islamabad 45320, Pakistan; b Department of Chemistry, University of Sargodah, Sargodah, Pakistan; c Department of Chemistry, Hong Kong Baptist University, Waterloo Road, Kowloon Tong, Hong Kong, People’s Republic of China

## Abstract

In the mol­ecule of the title compound, C_14_H_9_Cl_2_NO, the planar indole ring system [with a maximum deviation of 0.020 (2) Å for the N atom] is oriented at a dihedral angle of 72.17 (3)° with respect to the phenyl ring. In the crystal structure, weak inter­molecular C—H⋯O hydrogen bonds link the mol­ecules. A weak C—H⋯π inter­action may further stabilize the structure.

## Related literature

For general background, see: Hibino & Choshi (2002 ▶); Somei & Yamada (2003 ▶); Popp (1977 ▶, 1984 ▶). For related structures, see: Chakraborty & Talapatra (1985 ▶); Chakraborty *et al.* (1985 ▶); De (1992 ▶); De & Kitagawa (1991*a* ▶,*b* ▶); Itai *et al.* (1978 ▶). For bond-length data, see: Allen *et al.* (1987 ▶).
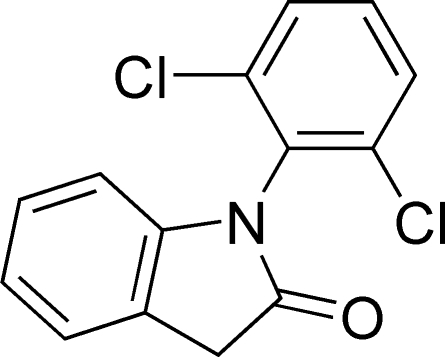


## Experimental

### 

#### Crystal data

C_14_H_9_Cl_2_NO
*M* *_r_* = 278.12
Monoclinic, 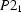

*a* = 7.1412 (8) Å
*b* = 8.0241 (9) Å
*c* = 11.0510 (13) Å
β = 105.789 (2)°
*V* = 609.35 (12) Å^3^
*Z* = 2
Mo *K*α radiation
μ = 0.52 mm^−1^
*T* = 173 K
0.30 × 0.24 × 0.20 mm

#### Data collection

Bruker SMART CCD area-detector diffractometer
Absorption correction: multi-scan (*SADABS*; Bruker, 2001 ▶) *T*_min_ = 0.755, *T*_max_ = 0.902
3710 measured reflections
2328 independent reflections
2295 reflections with *I* > 2σ(*I*)
*R*_int_ = 0.014

#### Refinement

*R*[*F* ^2^ > 2σ(*F* ^2^)] = 0.021
*wR*(*F* ^2^) = 0.060
*S* = 1.06
2328 reflections
163 parameters
1 restraint
H-atom parameters constrained
Δρ_max_ = 0.19 e Å^−3^
Δρ_min_ = −0.16 e Å^−3^
Absolute structure: Flack (1983 ▶), 705 Friedel pairs
Flack parameter: −0.02 (4)

### 

Data collection: *SMART* (Bruker, 2001 ▶); cell refinement: *SAINT* (Bruker, 2002 ▶); data reduction: *SAINT*; program(s) used to solve structure: *SHELXS97* (Sheldrick, 2008 ▶); program(s) used to refine structure: *SHELXL97* (Sheldrick, 2008 ▶); molecular graphics: *ORTEP-3 for Windows* (Farrugia, 1997 ▶) and *PLATON* (Spek, 2009 ▶); software used to prepare material for publication: *SHELXTL* (Sheldrick, 2008 ▶) and *PLATON*.

## Supplementary Material

Crystal structure: contains datablocks I, global. DOI: 10.1107/S1600536809005807/hk2625sup1.cif

Structure factors: contains datablocks I. DOI: 10.1107/S1600536809005807/hk2625Isup2.hkl

Additional supplementary materials:  crystallographic information; 3D view; checkCIF report

## Figures and Tables

**Table 1:** Hydrogen-bond geometry (Å, °)

*D*—H⋯*A*	*D*—H	H⋯*A*	*D*⋯*A*	*D*—H⋯*A*
C4—H4*A*⋯O1^i^	0.95	2.55	3.2267 (19)	128
C8—H8*A*⋯*Cg*1^ii^	0.99	2.74	3.6125 (23)	147

Symmetry codes: (i) 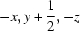; (ii) 
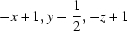
. *Cg*1 is the centroid of the C9–C14 ring.

**Fig. 1.**
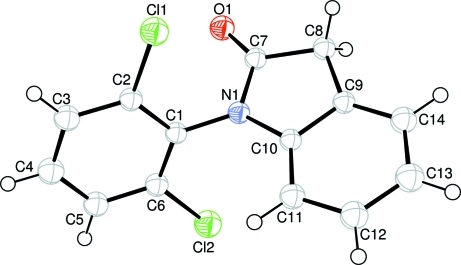
The molecular structure of the title molecule, with the atom-numbering scheme.

**Fig. 2.**
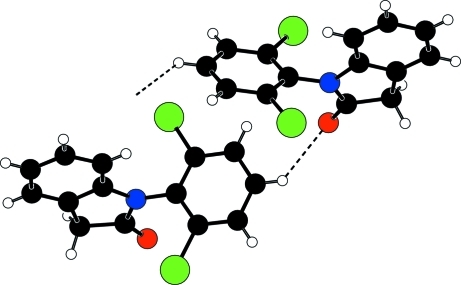
A partial packing diagram of the title compound. Hydrogen bonds are shown as dashed lines.

**Fig. 3.**
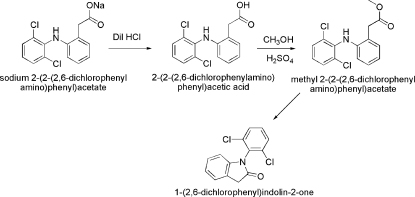
The formation of the title compound.

## supplementary crystallographic information

### Comment

Indolinones are a class of heterocyclic compounds found in many natural products and in a number of marketed drugs (Hibino & Choshi, 2002; Somei & Yamada, 2003). They have diverse chemical structures and complex physiological and pharmacological actions. The search for potential drugs and their mechanism of action has been difficult because of their complexity. These compounds contain both oxoindole and dioxolane moieties which have independently been seen in other anticonvulsants (Popp, 1977, 1984). The title compound, a chloro analogue, was found to be most potent in the MES test. Since no common target site has yet been established, X-ray analysis was undertaken to search its crystal structure, which may help to understand the mechanism of action at the molecular level.

In the title compound (Fig. 1), the bond lengths (Allen *et al.*, 1987) and angles are within normal ranges. Rings A (C1-C6), B (N1/C7-C10) and C (C9-C14) are, of course, planar and the dihedral angles between them are A/B = 71.73 (3)°, A/C = 72.43 (3)° and B/C = 1.07 (3)°. So, rings B and C are nearly coplanar. Ring A is oriented with respect to the planar indole ring system at a dihedral angle of 72.17 (3)°. The C8-C9 [1.4955 (19) Å] bond length may be compared with the corresponding values in other indoline nuclei (Itai *et al.*, 1978; Chakraborty & Talapatra, 1985; Chakraborty *et al.*, 1985; De & Kitagawa, 1991*a*,b; De, 1992).

In the crystal structure, weak intermolecular C-H···O hydrogen bonds (Table 1) link the molecules (Fig. 2), in which they may be effective in the stabilization of the structure. The weak C—H···π interaction (Table 1) may further stabilize the structure.

### Experimental

For the preparation of the title compound, sodium salt of 2-(2-(2,6-dichloro- phenylamino)phenyl)acetate (3.18 g, 10 mmol) was dissolved in distilled water (50 ml) and heated on a hot plate, until a homogeneous solution obtained, and then filtered to remove the undissolved product. It was poured into concentrated hydrochloric acid (5 ml) diluted with ice water (25 ml) in an Erlenmeyer flask to obtain 2-(2-(2,6-dichlorophenylamino)phenyl)acetic acid. Then, it was stand for 15 min in an ice bath. The crude product was separated and recrystallized in ethanol. 2-(2-(2,6-dichlorophenylamino)phenyl)acetic acid (2.96 g, 10 mmol) was refluxed in methanol (50 ml) in catalytic amount of sulfuric acid. As soon as a methyl ester is formed, it is cyclized to form the title compound, which was recrystallized in ethanol (yield; 79%; m.p. 420-421 K).

### Refinement

H atoms were positioned geometrically, with C-H = 0.95 and 0.99 Å for aromatic, and methylene H, respectively, and constrained to ride on their parent atoms, with U_iso_(H) = 1.2U_eq_(C).

### Crystal data

**Table:**

C_14_H_9_Cl_2_NO	*F*(000) = 284
*M**_r_* = 278.12	*D*_x_ = 1.516 Mg m^−^^3^
Monoclinic, *P*2_1_	Mo *K*α radiation, λ = 0.71073 Å
Hall symbol: P 2yb	Cell parameters from 2148 reflections
*a* = 7.1412 (8) Å	θ = 5.2–24.3°
*b* = 8.0241 (9) Å	µ = 0.52 mm^−^^1^
*c* = 11.0510 (13) Å	*T* = 173 K
β = 105.789 (2)°	Block, yellow
*V* = 609.35 (12) Å^3^	0.30 × 0.24 × 0.20 mm
*Z* = 2	

### Data collection

**Table:**

Bruker SMART CCD area-detector diffractometer	2328 independent reflections
Radiation source: fine-focus sealed tube	2295 reflections with *I* > 2σ(*I*)
graphite	*R*_int_ = 0.014
ω and φ scans	θ_max_ = 28.3°, θ_min_ = 3.1°
Absorption correction: multi-scan (*SADABS*; Bruker, 2001)	*h* = −9→9
*T*_min_ = 0.755, *T*_max_ = 0.902	*k* = −9→10
3710 measured reflections	*l* = −14→11

### Refinement

**Table:**

Refinement on *F*^2^	Secondary atom site location: difference Fourier map
Least-squares matrix: full	Hydrogen site location: inferred from neighbouring sites
*R*[*F*^2^ > 2σ(*F*^2^)] = 0.021	H-atom parameters constrained
*wR*(*F*^2^) = 0.060	*w* = 1/[σ^2^(*F*_o_^2^) + (0.0379*P*)^2^ + 0.0681*P*] where *P* = (*F*_o_^2^ + 2*F*_c_^2^)/3
*S* = 1.06	(Δ/σ)_max_ < 0.001
2328 reflections	Δρ_max_ = 0.19 e Å^−^^3^
163 parameters	Δρ_min_ = −0.16 e Å^−^^3^
1 restraint	Absolute structure: Flack (1983), 705 Friedel pairs
Primary atom site location: structure-invariant direct methods	Flack parameter: −0.02 (4)

### Special details

**Table:**

Geometry. All e.s.d.'s (except the e.s.d. in the dihedral angle between two l.s. planes) are estimated using the full covariance matrix. The cell e.s.d.'s are taken into account individually in the estimation of e.s.d.'s in distances, angles and torsion angles; correlations between e.s.d.'s in cell parameters are only used when they are defined by crystal symmetry. An approximate (isotropic) treatment of cell e.s.d.'s is used for estimating e.s.d.'s involving l.s. planes.
Refinement. Refinement of *F*^2^ against ALL reflections. The weighted *R*-factor *wR* and goodness of fit *S* are based on *F*^2^, conventional *R*-factors *R* are based on *F*, with *F* set to zero for negative *F*^2^. The threshold expression of *F*^2^ > σ(*F*^2^) is used only for calculating *R*-factors(gt) *etc*. and is not relevant to the choice of reflections for refinement. *R*-factors based on *F*^2^ are statistically about twice as large as those based on *F*, and *R*- factors based on ALL data will be even larger.

### Fractional atomic coordinates and isotropic or equivalent isotropic displacement parameters (Å^2^)

**Table:**

	*x*	*y*	*z*	*U*_iso_*/*U*_eq_	
Cl1	0.57381 (5)	0.76042 (5)	0.02255 (3)	0.03458 (10)	
Cl2	0.05095 (6)	0.97960 (6)	0.25885 (3)	0.03843 (11)	
O1	0.41005 (16)	0.54202 (14)	0.22865 (9)	0.0304 (2)	
N1	0.43177 (17)	0.82839 (15)	0.24614 (10)	0.0230 (2)	
C1	0.2984 (2)	0.86900 (18)	0.12903 (12)	0.0224 (2)	
C2	0.3471 (2)	0.83882 (17)	0.01693 (12)	0.0243 (3)	
C3	0.2172 (2)	0.8739 (2)	−0.09901 (13)	0.0302 (3)	
H3A	0.2512	0.8509	−0.1747	0.036*	
C4	0.0386 (2)	0.9425 (2)	−0.10285 (13)	0.0330 (3)	
H4A	−0.0507	0.9663	−0.1819	0.040*	
C5	−0.0126 (2)	0.9774 (2)	0.00703 (14)	0.0304 (3)	
H5A	−0.1350	1.0266	0.0036	0.036*	
C6	0.1176 (2)	0.93932 (19)	0.12198 (12)	0.0266 (3)	
C7	0.48390 (19)	0.66561 (18)	0.28346 (12)	0.0233 (3)	
C8	0.6499 (2)	0.67599 (18)	0.40391 (12)	0.0249 (3)	
H8A	0.6135	0.6223	0.4749	0.030*	
H8B	0.7688	0.6219	0.3929	0.030*	
C9	0.6805 (2)	0.85913 (18)	0.42570 (12)	0.0234 (3)	
C10	0.54970 (19)	0.94523 (17)	0.32861 (11)	0.0219 (2)	
C11	0.5448 (2)	1.11669 (19)	0.32044 (13)	0.0286 (3)	
H11A	0.4558	1.1731	0.2533	0.034*	
C12	0.6774 (2)	1.2032 (2)	0.41607 (14)	0.0335 (3)	
H12A	0.6783	1.3216	0.4138	0.040*	
C13	0.8073 (2)	1.1219 (2)	0.51397 (15)	0.0357 (3)	
H13A	0.8950	1.1846	0.5778	0.043*	
C14	0.8101 (2)	0.9477 (2)	0.51951 (13)	0.0310 (3)	
H14A	0.8994	0.8912	0.5865	0.037*	

### Atomic displacement parameters (Å^2^)

**Table:**

	*U*^11^	*U*^22^	*U*^33^	*U*^12^	*U*^13^	*U*^23^
Cl1	0.03021 (17)	0.0422 (2)	0.03333 (16)	0.00882 (15)	0.01213 (12)	−0.00025 (14)
Cl2	0.04088 (19)	0.0480 (2)	0.03094 (16)	0.01679 (17)	0.01741 (14)	0.00795 (15)
O1	0.0330 (5)	0.0230 (5)	0.0309 (5)	−0.0025 (4)	0.0011 (4)	−0.0026 (4)
N1	0.0249 (5)	0.0208 (5)	0.0204 (5)	0.0012 (4)	0.0010 (4)	−0.0009 (4)
C1	0.0235 (6)	0.0220 (6)	0.0197 (5)	0.0010 (5)	0.0025 (5)	0.0010 (4)
C2	0.0245 (6)	0.0223 (6)	0.0256 (6)	0.0011 (5)	0.0063 (5)	−0.0021 (5)
C3	0.0358 (8)	0.0322 (8)	0.0206 (6)	0.0009 (6)	0.0043 (5)	−0.0003 (5)
C4	0.0320 (7)	0.0379 (8)	0.0240 (6)	0.0021 (6)	−0.0012 (5)	0.0041 (6)
C5	0.0242 (6)	0.0343 (8)	0.0303 (6)	0.0058 (6)	0.0035 (5)	0.0062 (6)
C6	0.0274 (7)	0.0276 (7)	0.0251 (6)	0.0031 (6)	0.0077 (5)	0.0034 (5)
C7	0.0216 (6)	0.0239 (6)	0.0229 (5)	0.0004 (5)	0.0037 (5)	0.0007 (4)
C8	0.0235 (6)	0.0236 (6)	0.0241 (6)	0.0037 (5)	0.0006 (5)	0.0007 (5)
C9	0.0224 (6)	0.0244 (7)	0.0226 (5)	0.0010 (5)	0.0049 (5)	−0.0020 (5)
C10	0.0236 (6)	0.0225 (6)	0.0194 (5)	−0.0006 (5)	0.0055 (4)	−0.0032 (4)
C11	0.0352 (8)	0.0236 (7)	0.0290 (6)	0.0008 (6)	0.0122 (6)	0.0003 (5)
C12	0.0397 (8)	0.0230 (7)	0.0414 (7)	−0.0057 (6)	0.0173 (7)	−0.0081 (6)
C13	0.0303 (8)	0.0373 (8)	0.0389 (8)	−0.0068 (7)	0.0085 (6)	−0.0162 (6)
C14	0.0259 (7)	0.0371 (8)	0.0272 (6)	−0.0002 (6)	0.0023 (5)	−0.0080 (5)

### Geometric parameters (Å, °)

**Table:**

C1—C6	1.392 (2)	C8—C9	1.4955 (19)
C1—C2	1.3958 (18)	C8—H8A	0.9900
C1—N1	1.4203 (15)	C8—H8B	0.9900
C2—C3	1.3908 (19)	C9—C14	1.3834 (19)
C2—Cl1	1.7223 (14)	C9—C10	1.3984 (18)
C3—C4	1.379 (2)	C10—C11	1.379 (2)
C3—H3A	0.9500	C10—N1	1.4146 (16)
C4—C5	1.389 (2)	C11—C12	1.397 (2)
C4—H4A	0.9500	C11—H11A	0.9500
C5—C6	1.3890 (19)	C12—C13	1.382 (2)
C5—H5A	0.9500	C12—H12A	0.9500
C6—Cl2	1.7353 (14)	C13—C14	1.399 (2)
C7—O1	1.2061 (17)	C13—H13A	0.9500
C7—N1	1.3896 (18)	C14—H14A	0.9500
C7—C8	1.5250 (17)		
			
C6—C1—C2	118.16 (12)	C9—C8—H8B	111.0
C6—C1—N1	121.73 (12)	C7—C8—H8B	111.0
C2—C1—N1	120.12 (12)	H8A—C8—H8B	109.0
C3—C2—C1	121.19 (13)	C14—C9—C10	119.49 (14)
C3—C2—Cl1	119.46 (11)	C14—C9—C8	131.59 (14)
C1—C2—Cl1	119.34 (10)	C10—C9—C8	108.91 (12)
C4—C3—C2	119.23 (13)	C11—C10—C9	122.89 (13)
C4—C3—H3A	120.4	C11—C10—N1	128.25 (13)
C2—C3—H3A	120.4	C9—C10—N1	108.86 (12)
C3—C4—C5	121.01 (13)	C10—C11—C12	116.53 (14)
C3—C4—H4A	119.5	C10—C11—H11A	121.7
C5—C4—H4A	119.5	C12—C11—H11A	121.7
C4—C5—C6	119.01 (14)	C13—C12—C11	121.99 (15)
C4—C5—H5A	120.5	C13—C12—H12A	119.0
C6—C5—H5A	120.5	C11—C12—H12A	119.0
C5—C6—C1	121.38 (12)	C12—C13—C14	120.31 (14)
C5—C6—Cl2	118.81 (11)	C12—C13—H13A	119.8
C1—C6—Cl2	119.81 (10)	C14—C13—H13A	119.8
O1—C7—N1	125.35 (11)	C9—C14—C13	118.77 (14)
O1—C7—C8	127.81 (13)	C9—C14—H14A	120.6
N1—C7—C8	106.83 (11)	C13—C14—H14A	120.6
C9—C8—C7	103.83 (11)	C7—N1—C10	111.54 (10)
C9—C8—H8A	111.0	C7—N1—C1	123.04 (11)
C7—C8—H8A	111.0	C10—N1—C1	124.68 (11)

### Hydrogen-bond geometry (Å, °)

**Table:**

*D*—H···*A*	*D*—H	H···*A*	*D*···*A*	*D*—H···*A*
C4—H4A···O1^i^	0.95	2.55	3.2267 (19)	128
C8—H8A···Cg1^ii^	0.99	2.74	3.6125 (23)	147

Symmetry codes: (i) −*x*, *y*+1/2, −*z*; (ii) −*x*+1, *y*−1/2, −*z*+1.
